# The Brightest Rainbow Follows the Darkest Storm

**DOI:** 10.3390/children7110223

**Published:** 2020-11-10

**Authors:** Douglas Lake

**Affiliations:** 1 Department of Radiology, McFarland Clinic, 1215 Duff Avenue, Ames, IA 50010, USA; douglakemd@gmail.com; 2 Department of Radiology, Stanford School of Medicine 300 Pasteur Drive, Palo Alto, CA 94301, USA

**Keywords:** pulmonary vein stenosis, hope

## Abstract

A parent’s perspective on pulmonary vein stenosis through the experience of two children with the disease.

## 1. Introduction

Caroline is our rainbow baby, born after the loss of another child, the light and color arising after a storm passes. A rainbow baby lifts a family in desperate need of lifting.

It is a beautiful thing that I hope you never experience.

## 2. Discussion

Caroline was born on 10 December 2015. Just three months later, my wife Maleia and I found ourselves trailing an emergency medical helicopter flying our daughter to Iowa City. No words can fully describe the emotional pain we felt as we drove in stunned silence. *Why is this happening again*? We had already lost more than anyone should have to bear.

Two years earlier, our son Ben, almost four months old and already greatly beloved by his two older sisters as well as his parents, was just beginning to laugh and smile. His tiny hands—hands that might someday hold mine crossing a street, wave out a school bus window, or toss a football—would squeeze my pinky finger. His “favorite” shirt featured a little red dragon on the front.

That shirt concealed a real fire-breathing monster just inside the tiny chest it covered. Over several weeks, Ben had drifted lower on the growth charts, and our pediatrician suggested admitting him to the local children’s hospital to figure out once and for all the best formula to keep him healthy and growing. We thought.

Ben rapidly declined in his first few days in the hospital. Increasingly intense testing revealed the truth: pulmonary vein stenosis (PVS). The same day the diagnosis was made, Ben died—21 March 2014.

*Pulmonary vein stenosis? In kids?* I did not learn about this in medical school, residency, or fellowship. Maybe in adults, after atrial fibrillation treatment, but *kids*? *Infants?*

Grief for Ben hit me hard. Everyday taken-for-granted events such as walking across a street were gone. First day of school pictures? Gone. Football weekends? Gone. When I lost my son, I also lost years of experiences I had already unconsciously imagined. Similar to a lot of parents of newborns, Maleia and I had divided responsibilities in order to cover everything, and I had primarily cared for our two older girls while Maleia primarily cared for Ben.

I had thought nothing of it—I had years to spend with Ben, right? The moments I had missed haunted me now.

Grief hit all of us hard, of course, if sometimes in different ways.

I found myself distracted at work, sometimes tracing images of pulmonary veins, and thinking about Ben. On my first day back to work, an ultrasound came for me to read—a right upper quadrant on a twenty-four-year-old named Benjamin. To this day, I am not sure how the hospital transcriptionists made out what I said. I cried through the entire dictation.

I beat myself up wondering if earlier intervention would have helped more. What good was all my medical training? Should I have not *known*? Should I have not been able to *do* something?

Grief would creep up when I least expected it. Simple everyday events became emotional minefields. When “Stay with Me” by Sam Smith or “On Top of The World” by Imagine Dragons came on the radio, it was more than I could stand, and I had to change the station.

Sometimes, I asked Maleia to stay in the bathroom and talk while I showered because the tears fell heaviest when water hit my head.

One day, I went for a haircut and chatted with the stylist about my two older girls. When she innocently asked, “So, are you going to try for a boy?” I slowly exhaled, told Ben’s story, and tried to ignore the emptiness in my chest.

My younger daughter began to cry during church at the same point in the service each week. “What’s the matter, sweetness?” I would ask. One day, she finally expressed her answer: “I miss Ben”.

My oldest daughter rode a grief rollercoaster through some painful loop-the-loops. It took us months to figure out that her “ups” were her normal, and her “downs” were expressions of her grief. We learned to stop at the Build-A-Bear Workshop any time we came near a mall. Making a wish, kissing the heart, and giving the bear life—this child-sized routine came to help our daughters process their grief. Each stuffed creation brought them a little closer to the brother they lost.

We adults needed a different place to turn to with our grief, and we found it at The Compassionate Friends, a support network for people grieving the loss of a child. I took strange comfort from seeing another parent tear up as she explained that she still grieved the loss of her child more than 20 years later. That assured me the hole in my heart would never completely heal—I did not want it to—and that I was not alone.

Something in medical training sucks empathy out of doctors. It is self-protective—you cannot let yourself go through everything all of your patients feel. When roles reversed and I was the parent in need of empathy, something in me shifted again. Having lost Ben, and with the help of Compassionate Friends in understanding and processing my grief, my own compassion has been rebuilt. Today, when I walk into a room to deliver a patient bad news, it only takes a second of thinking back to any number of events with Ben or Caroline for the empathy to just flow out.

This is where our family was when Caroline was born, and she brought our family joy we desperately needed. We thought the absolute worst of the storm was passing. However, really, we were in the eye of the hurricane. A fierce storm still raged just behind our backs.

The first time Caroline threw up formula, dad-me thought, “*Oh no! That’s what Ben did*”.

Logical, evidence-based doctor-me thought, “PVS is a 1-in-1-million diagnosis. It’s not an inherited disease. All kids spit-up formula, Doug.”

As time passed, Caroline drifted down the growth curve. Our pediatrician urged us to do an echocardiogram and a chest X-ray.

As a radiologist, I will never forget seeing Caroline’s chest X-ray. Normal sized heart—and the ugliest pulmonary edema pattern imaginable. In 18 years and tens of thousands of chest X-rays, I had only seen this pattern once.

Ben.

I did not need the echo (echocardiogram), CTA (computed tomography angiography), or cardiac catheterization to tell me. I knew.

PVS.

Shock, sadness, fear…words fail. Grief roared back, a category five hurricane. I did not feel anything. I cried, but the hollowness inside me was the worst. Even four years later, it is hard to describe the brutality of that emotional emptiness.

There was no time right then, with the X-ray and testing, to process much about the experience we were suddenly thrown into. There was too much to do. Decisions had to be made now that it was clear my daughter, my family, and even my community, would have to fight this dragon.

How bad was Caroline’s PVS? Could it be something else? Do we even have a week? Should we transfer her to Des Moines or Iowa City? How could we even begin to explain to our older girls, amid their existing grief?

Anxiety, sadness, and fear dominated our thoughts while we decided on the University of Iowa and followed our tiny rainbow to a larger hospital in search of any ray of hope.

Hope was in short supply, though. University of Iowa pediatric cardiologists confirmed PVS and offered two choices:

Simply love on Caroline for another month. Two at the most.

Or,

Take a chance and transfer her to one of only a handful of hospitals in the world that attempt to treat PVS in children (Boston Children’s Hospital).

We chose choice two AND loved on her as hard as we could. Every. Single. Month.

That is where Caroline’s story diverges from Ben’s. Caroline, too, needed massive support in the cardiac ICU (intensive care unit). She came in on a Sunday morning and was an emergency add-on case by Monday morning. They found severe disease in three veins, with some atretic veins. One vein somehow kept Caroline alive. Balloons opened her veins and were a rickety, unstable bridge that got Caroline to cardiac surgery a week later. Many medicines—imatinib, bevacizumab, sildenafil, sirolimus, losartan, Lasix—became part of Caroline’s life.

We celebrated Caroline’s four-month birthday. Then her five-month, and her six-month. We did not know how many of these we would get—we were not at all sure she would reach her first birthday.

However, we did. Disease recurrences slammed our still-recovering home similar to hurricane rainbands, setting everyone on edge. We lived with the fear that we were only one bad test or procedure from our storm resurging. Maleia kept an emergency “hospital bag” packed and ready to go for when Caroline needed to return to the hospital. We nearly lost Caroline during an emergency evacuation flight to Boston. For two very difficult years, our family lived split between two cities, to keep Caroline within a mile of Boston Children’s Hospital.

It worked. Our storm surges gradually lessened and PVS passed.

Preparing for Caroline’s last cardiac catheterization, we had a first. Her heart got … SMALLER! Rather than 1.2× or 1.5× systemic pressures in her right heart, she is closer to “normal” than ever.

Best of all, we are finally back together as a family, living under one roof.

Caroline is “almost five” years old—her words. She swings similar to a monkey from a gymnastic beam in our home, builds Lego creations, chases her sisters, and started remote preschool this fall.

## 3. Conclusions

This special edition of Children devoted to PVS is edited by Caroline’s pediatric cardiologist and chief dragon-slayer, Dr. Kathy Jenkins. I applaud Children for taking on the subject, and thank Dr. Jenkins and Drs. Ryan Callahan, Chris Baird, John Kheir, and Jim Lock, and nurse practitioner Christina Ireland, and so many professionals pushing back against this previously lethal disease for children and families around the world. Targeted therapy to suppress the myofibroblast has changed the 0% survival of 20 years ago into 77% survival today [1].

Challenges remain. Is there an optimal treatment pathway? What role do the various medications play in suppressing the myofibroblast? How do you best support families navigating their category five hurricane?

Thank you to all who help children and their families face this challenging and complex disease daily. Your dedication and continual push for progress has produced the advances that have saved my daughter’s life. Saved my family. Saved *me*.
